# Developmental trajectories of gambling severity after cognitive-behavioral therapy

**DOI:** 10.1192/j.eurpsy.2022.935

**Published:** 2022-09-01

**Authors:** S. Jimenez-Murcia, R. Granero, F. Fernandez-Aranda

**Affiliations:** 1 Bellvitge University Hospital-IDIBELL and CIBERobn, Psychiatry Department, Hospitalet del Llobregat, Spain; 2 Autonomous University of Barcelona, Department Of Psychobiology And Methodology, Bellaterra, Spain; 3 University Hospital of Bellvitge-IDIBELL and CIBERobn, Department Of Psychiatry, Barcelona, Spain

**Keywords:** personality, gambling, predictors, Psychotherapy

## Abstract

**Introduction:**

Gambling disorder (GD) is characterized by repeated problematic gambling behavior associated with unsuccessful and uncontrollable urges to keep gambling, which leads to considerable distress and impairment. Several types of interventions exist to treat GD, with cognitive behavior therapy (CBT) being one of the most widely used approaches.

**Objectives:**

To estimate trajectories of the gambling disorder (GD) severity for 12 months following a manualized cognitive-behavior-therapy (CBT) program, and to identify the main variables associated with each trajectory.

**Methods:**

Latent Class Growth Analysis examined the longitudinal changes of n = 603 treatment-seeking patients with GD.

**Results:**

Five separate empirical trajectories were identified: T1 (n = 383, 63.5%) was characterized by the most highest baseline gambling severity levels and positive progress to recovery during the follow-up period; T2 (n = 154, 25.5%) featured participants with high baseline gambling severity and good progress to recovery; T3 (n = 30, 5.0%) was made up of patients with high gambling baseline severity and slow progress to recovery; T4 (n = 13, 2.2%) and T5 (n = 23, 3.8%) contained participants with high baseline gambling severity and moderate (T4) and poor (T5) progress in GD severity during the follow-up. Psychopathology, personality traits, poor compliance and relapses discriminated between trajectories.

**Conclusions:**

These results show that treatment seeking patients with GD are heterogeneous. In addition, the obtained findings could be useful in the design of more efficient interventions for this behavioral addiction. Funding oftained from RTI2018-101837B-I00

**Disclosure:**

No significant relationships.

